# Our “Chesterfield” Again

**Published:** 1872-06

**Authors:** 


### PART III.

### Editorials, Correspondence and Miscellaneous.

### "OUR CHESTERFIELD" AGAIN.

The editor of the "largest medical monthly in America," since the announcement made that THE COMPANION had met with a success unprecedented in the history of medical journalism South, has evinced no little animosity towards this journal and its editors.

Drawn thus into a controversy not of our seeking; assaulted with deliberation and design; taunted, and charged with charlatanism in the conduct of our journal; with private character assailed and denounced as "stained;" our associates declared to have been deceitfully entrapped by us, we are forced to a vindication, and compelled to assume the task of self defense.

Why is all this? Can our readers imagine any other cause save the success of THE COMPANION? It would seem that the editor of the "largest medical monthly in America" holds Southern territory as a "divine right" for his journal. Any interference with this claim calls forth the strongest expressions of anger and vituperation. His alarm may be well founded. Fearing this, he bitterly assailed the management of this journal, and endeavored to destroy its influence by charging upon the editors "a want of good taste, ethics, propriety and decorum."

Foiled in this, and compelled to gulp down a most nauseous compound of his own manufacturing—having been shown to be the delectable subject of his own pen-painting; a boaster, a violator of good taste, and a bundle of inconsistencies—he spikes his feeble guns, refuses to support his denunciations of The Companion by an appeal to the ethics, and—lost in the mazes of his own reckless folly—strikes madly, like an adder, at the private character of the "chief editor" of this journal. We commiserate the straits of so bold an accuser—the mishaps of so self-confident an acrobat. But while pitying the utter wreck of his hopes under the spell of the "green-eyed monster"—who, it seems, claims his victims from the domain of journalism no less than from the realms of love—the ludicrousness of his self-inflicted punishment calls for merriment rather than anger.

"The largest medical monthly in America"—a professional "sky-lifting" for a pecuniary consideration, as the "proprietor" of a "commercial" commodity—is surely in *articulo mortis!* Its usual richness of metaphor, and wonted appeal to facts as the summit of its rhetoric, are sorely at fault when, in lieu of an argument, it presents its readers a diatribe upon the assumed ethical tergiversations of the "chief editor" of The Companion.

Our Chesterfield, among his other self-inspired vagaries, prides himself upon his subtle critical acumen as a reviewer. Criticism is his chosen field, as it is of all pretenders to sublimity of intellect and infallibility of judgment. This "mental constitution" rests solely upon an assumacy of such powers—self-blown into a pigmy Pope, without "Cardinal or Church." Thus, self-endowed, with a strut and swell peculiarly his own, he enacts the role, not only of the "reviewer," but the antiquarian as well. He has found a lost document. With critical ardor he resurrects it, and grows quixotically enthused over the discovery "of many facts hitherto concealed from the public" in that veracious "exposition" of distortions and one-sided assertions, dignified by being styled "a statement of facts."

The "facts"—ye Gods hide your heads!—so gallantly brought to view by the medical Pope and hypercritical "reviewer," like good wine, are old; but that suits the purpose just as well to locate his journalistic spite in consideration of the jostlings the "yearling" has given "the largest medical monthly in America."

"A statement of facts," so called, has gained much in popish opinion after one year's repose in the mausoleum of forgetfulness. He now, illumined by our success, discovers a marvellous value in the dusty pamphlet he quietly read, but did not review, just one year ago. And forgetful of his Pecksniffian platitudes in the same issue, wherein it is affirmed that "if a reviewer could so far forget his self respect as to stoop to envy, jealousy, or any unworthy feeling, in the declaration of his opinions, he ought" to be pitied rather than reprobated, he rubs away the dust from the moulding covers of a "statement of facts," and impales himself upon his own lance. The "statement of facts" was old and mellow—a grand, gushing, "hopeful," without name or parentage—having not only met his eye one year ago, but his critical vision in the college organ. Not only so, but other pamphlets containing "facts" drawn from the records, and having the sanction of names, were placed in his hands. These he has not "reviewed," and never will—that would not carry out the "chief" design of crushing THE COMPANION.

Our "reviewer," lost to every motive save one, the most "despicable" of all, and oblivious of his own words, "goes back" upon himself and his Georgia friends. To gratify his spleen, he seeks to justify the old faculty of the Atlanta Medical College, by assuming "a statement of facts" to be true, thus stigmatizing the professional gentleman of Macon, Savannah, and other places, as well as three annual meetings of the Georgia Medical Association, in his quixotic attempt to "stain" the character of the "chief editor" of THE COMPANION. He not only, as stated, "goes back" on his friends in Georgia, but on himself. Last

September, after being twice put in possession of "a statement of facts," so called, with counter statements made by the Fulton County Medical Society, Macon Medical Association, and Georgia Medical Society, of Savannah, he then asserted: "*So far as can be ascertained here, the Faculty of the Atlanta Medical College is generally and justly censured for its course!!!*"

Strange to say, with no additional source of information, save the unprecedented success of THE COMPANION, he now, after peace has been declared in Georgia upon terms laid down by the opponents of "a statement of facts," turns "reviewer," and attempts to re-open an old sore, and at the same time stultifies his record by asserting that a "statement of facts" is "an interesting (very, surely) exposition of *many* facts (please remember, gentlemen of the Georgia Medical Association) hitherto *concealed* (by the reviewer himself, from his readers) from the public."

The "chief editor" of THE COMPANION has invoked and demanded the most exhaustive scrutiny of all charges against him — having branded them as false and slanderous. While a member of the Faculty of the Atlanta Medical College, a portion of the Faculty, contrary to ethical law, and without the consent or knowledge of the Trustees, procured an amendment to the charter which the Trustees denounced as unconstitutional. This being reported to the Georgia Medical Association, was declared to confer upon the Faculty "unusual and extraordinary powers," and in consequence thereof the College was declared "irregular." Two of his colleagues, with four professors of their appointment, (against the wishes of the Trustees) sought to depose the "chief editor" from his chair under the powers of the amendment, which by the advice of the Trustees, he resisted. In order to furnish a pretext—the real motive being, as the facts will prove, his opposition to the illegal manner by which the amendment was procured, the unprofessional and unscriptural privileges it granted the Faculty, and the unsatisfactory management of the finances —a resolution was voted by the above, in which he was requested to resign (in order to cover up their wrongs by assailing their opponent) in which was offered as a reason his scheme of erecting a "Ladies Hospital;" a scheme which the profession had indorsed, as well as four of the six members of the old Faculty. So untenable was the charge (so called) that every member of the original Faculty, save two, refused to vote.

The Trustees demanded written charges, with proof to sustain them. This the Faculty was unable to furnish, and in defiance of the Board, cut themselves loose, ran the College, and graduated students independent of it.

It is not our wish to write a history of this controversy here; that has already been done. We deeply regret the necessity of referring to transactions we hoped, for the honor of the profession of Georgia, had been buried from public view. Yet, as our "reviewer" hopes to bring discredit upon this journal, and thus check its progress by alienating our associates and readers, we are left no other alternative but to resurrect the past. That this is the real motive of our reviewer is apparent. Defeated on the first line of assault, he now, with pharisaical sanctity, asserts that "it is only wonderful that one so stained can have associated with him editorially the number of estimable (having previously declared their connection an imposition !) gentlemen whose names occur on the cover of that journal." And continues: "There is but one explanation of such a misfortune (doubtless to the "largest medical monthly in America") and this is that the physicians whose names appear are ignorant of the facts set forth officially (without a name !) in the pamphlet under review."

Our reviewer thus performs a gastronomical feat, similar to his prototypes of old, of "straining at a gnat and swallowing a camel." He sees much in Dr. Yandell, with his *home* reputation and flourishing institution, to cause the most horrid distortions of countenance in his "straining," while, without effort, he gulps down, at one mouthful, the old Faculty of the Atlanta Medical College, while in open rebellion to the Trustees of the institution, the Code of Ethics, and the Georgia Medical Association ! And all for the highly creditable object of stabbing a journal which makes the "proprietor" of "the largest medical monthly in America" quake in his boots.

The "chief editor" does not fear such assaults. His associates and friends are not automatons to be moved hither and thither at the behest of an ungenerous and envious "reviewer."

But in respect to them, and in order that they may see how much truth there is in the charge gratuitously made, that they have allied themselves to the "chief editor" who is "stained" in character, he appeals to facts within the reach of all.

He has been indorsed fully and completely—first, by the old (and new) Board of the Atlanta Medical College, after the so called charges were preferred. The following is taken from the recorded proceedings of the Board:

WHEREAS, Dr. T. S. Powell was, by the Faculty of the Atlanta Medical College, invited to resign his chair as Professor of Obstetrics and Diseases of Women and Children, which he declined to do, appealing to the Board of Trustees, and whereas, said Board of Trustees having called upon said Faculty for charges, specifications and proof against Dr. Powell, which they have failed to furnish; therefore

*Resolved*, That we know nothing against Dr. Powell, either of our own knowledge, by report, or otherwise, derogatory to his character, standing, or ability as a gentleman, scholar, or professor, and take pleasure in adding our testimony in behalf of his ability, honor, integrity, and efficiency, and *earnestly request* that he shall retain his position in said College, and continue, as heretofore, faithfully and skillfully to perform the high trust reposed in him; which was adopted.

Second, he has been sustained by his Society, the Fulton County Medical Society. The committee appointed for the purpose of arriving at the truth, in their report, which was adopted and published, say:

The statement of facts contained in the foregoing defense of Dr. Powell, beginning with the difficulty which grew out of Dr. Westmoreland's management of the finances of the College, and ending with the attempted expulsion of Dr. Powell from the Faculty, is a complete and triumphant vindication of his conduct and character. It presents him as an injured man, against whom grave accusations have been made, but seeking in vain that investigation at the proper tribunal. This was denied him by his accusers; the challenge for an inquiry into the truth of the charges which they made against him was declined by them; and, according to all rules of justice, he stands acquitted of these charges, before the public as well as the profession. In his opposition to the scheme to amend the charter of the College, he no doubt gave offense to the Faculty, but in this he has been sustained by the action of the Legislature, in repealing the amendment, by the action of the Board of Trustees, and by the Georgia Medical Association. But as your Committee have hereinbefore stated, the unprofessional conduct which the Faculty imputed to him, and which was made the ground of their action against him, was his connection with the enterprise known as the "Ladies' Home," a literary journal published in the city of Atlanta. Your Committee have recently examined the files of that journal with sufficient attention and care to enable them to form a judgment of its true character, and they found in its pages nothing whatever that can be made the foundation of the charge preferred against Dr. Powell, because of his connection with it. On the contrary, it is a work of rare literary merit, is pervaded by a high moral tone, and reflects credit upon its conductors and contributors.

Third, he has been sustained by the actions of three annual meetings of the Georgia Medical Association.

Fourth, the Faculty of the Atlanta Medical College have admitted the falsity of the charges themselves, in that, after the charges were made by which our reviewer asserts the "chief editor" was stained, the Faculty did, upon their own motion, propose to "rescind all action heretofore had" against him, and tendered him, through the Trustees, the position of "Emeritus Professor," as will be seen from the following:

*To the President of the Board of Trustees of the Atlanta Medical College:*

The Faculty of the institution under your supervision, being earnestly desirous of terminating amicably the difference existing between them and the Trustees, without further litigation, beg leave to submit, through you to the Board, that you represent the following basis of settlement, viz:

1. That the original charter of the College, and the amendment thereto, be superceded by a new charter, to be obtained as early as practicable, which shall be mutually acceptable to the Trustees and Faculty.

2. That until such charter is obtained, the Faculty shall have the power of nominating proper persons to fill all vacancies which may occur, and the Trustees the power of approving or rejecting such nominations.

3. That the Faculty shall consist, until altered by the future action of the Faculty and Trustees, of seven regular Professors, and one Emeritus Professor, as follows, viz:

Thomas S. Powell, M. D., Emeritus Professor.

A. Means, M. D., Professor of Medical and General Chemistry.

D. C. O'Keefe, M. D., Professor of Theory and Practice of Medicine.

W. F. Westmoreland, M. D., Professor of Principles and Practice of Surgery.

H. V. M. Miller, M. D., Professor of Obstetrics and Diseases of Women and Children.

Ebin Hillyer, M. D., Professor of Institutes of Medicine.

S. H. Stout, M. D., Professor of Anatomy.

J. G. Westmoreland, M. D., Professor of Materia Medica and Therapeutics.

4. As a part of this settlement, it is understood that the Faculty rescind all action heretofore had in relation to Professor T. S. Powell.

A. Means,
Chairman Faculty Atlanta Medical College.

All this was in possession of the "reviewer" had he chosen to have availed himself of it.

Having accomplished our purpose, and proven the assertions of our reviewer to be false, it only remains to be seen if that justice and fair dealing which he so prides himself upon shall bring the correction.

In the meantime, we assure our friends no sort of persecution shall drive The Companion into a personal controversy beyond the absolute necessity of defending its reputation and the character of its editors. We therefore for the present dismiss our accuser, with all the odium of having inaugurated a warfare against us for no better purpose, it is believed, than the achievement of pecuniary success, in behalf and in consideration of the "proprietor" of "the largest medical monthly in America." If, however, he feels so tenderly for, and admires so greatly, the old Faculty of the Atlanta Medical College as to indorse their past record, and to proclaim himself the champion of their errors, we do not object. Should he, under the spell of a new-born friendship, and the sympathetic drawings of that "fellow feeling" which makes such allies "wondrous kind," desire to discuss the principles maintained by his newly chosen friends he seems now so anxious to indorse, being thus forced, we accept the challenge, and appealing to truth and ethics alone, defy him to "stain" the professional record or honor of the "chief editor" of The Companion.

